# Fracture Resistance of Mineral Trioxide Aggregate- and Biodentine-Pulpotomized teeth with Different Restorative Techniques: An in vitro Study 

**DOI:** 10.30476/dentjods.2025.105152.2573

**Published:** 2026-06-01

**Authors:** Hajar Farhadpour, Yasamin Ghahramani, Mohammadhossein Jahani, Hanie Poursoleyman, Mahshid Mohammadi-Bassir

**Affiliations:** 1 Oral and Dental Disease Research Center, Dept. of Operative Dentistry, School of Dentistry, Shiraz University of Medical Sciences, Shiraz, Iran.; 2 Dept. of Endodontic, School of Dentistry, Shiraz University of Medical Sciences, Shiraz, Iran.; 3 Postgraduated Resident Dept. of Periodontics, School of Dentistry, Shiraz University of Medical Sciences, Shiraz, Iran.; 4 Dentist, Shiraz, Iran.; 5 Dept. of Operative Dentistry, School of Dentistry, Shahed University, Tehran, Iran.

**Keywords:** Fracture Strength, MTA, Biodentine, Pulpotomy, Dental Restoration

## Abstract

**Background:**

Preserving the radicular pulp in traumatically or mechanically exposed teeth through coronal pulpotomy (CP) is an advantageous endodontic procedure.

**Purpose:**

This study compared the fracture resistance (FR) of mineral trioxide aggregate (MTA) and Biodentine (BD) in pulpotomized maxillary premolars restored with different materials.

**Materials and Method:**

Ninety extracted maxillary premolars were divided into 9 groups (n=10). Group 1 served as intact controls. For the other groups, after access cavity preparation, half of the teeth were pulpotomized with MTA, and the others with BD. Groups 2 (MTA/unrestored) and 3 (BD/unrestored) remained unrestored. Restorations were as follows: Groups 4 (MTA/GI+Am) and 5 (BD/GI+Am) were restored with glass ionomer and amalgam; groups 6 (MTA/RMGI+ conventional com) and 7 (BD/RMGI+conventional com) with resin-modified glass-ionomer and conventional composite resin; groups 8 (MTA/bulk-fill com) and 9 (BD/bulk-fill com) with bulk-fill composite. After 24 hours, the FR test was conducted. Data were analyzed by one-way ANOVA and the Tukey test (*p* Value< 0.05).

**Results:**

FR of control group was significantly higher than the all other groups (*p*> 0.05). There was no significant difference between two unrestored MTA and BD groups (2 and 3) (p= 0.62). Group 2 (MTA/unrestored) showed significantly lower FR compared to all composite restored groups (6-9) (*p*< 0.05), and comparable to amalgam restored groups (4 and 5). The higher FR of all the restored groups (4-9) compared to BD/unrestored group (group 3) was not significant *p*> 0.05). There was no significant difference between all the restored groups in terms of FR and fracture type rate (*p*.> 0.05).

**Conclusion:**

Both composite resins increased the strength of MTA-pulpotomized premolars, but this was not the case for BD. FR of amalgam restorations did not significantly differ from both composite resin restorations. However, none of the tested restorations was capable to recover the fracture strength completely.

## Introduction

Preserving the vital radicular pulp in teeth that are traumatically injured or mechanically exposed through coronal pulpotomy (CP) is an advantageous endodontic procedure. This method offers the benefit of stress absorption and proprioceptive functions of the radicular pulp, which helps in preventing overloading and fractures. Additionally, CP is less technically demanding, quicker, and more cost-effective than root canal treatment [ 1 ]. It is commonly used in immature teeth as a regenerative endodontic procedure to encourage normal root apex maturation [ 2 ]. Several studies have shown that CP is highly successful in treating traumatic and cariously exposed permanent teeth with closed root apices [ 2 - 5 ]. A survival analysis of permanent teeth treated with pulpotomy indicated that the age range (8 to 79 years) of the treated teeth did not significantly impact the success rate [ 6 - 7 ].

Mineral trioxide aggregate (MTA) and Biodentine (BD) are two types of calcium silicate-based cements used as pulpotomy agents. They possess dentin-like mechanical properties and positively affect vital pulp cells, stimulating tertiary dentin formation [ 8 ]. BD, in particular, offers easier handling, lower cost, and quicker setting times, making it more convenient than MTA. Studies have also shown that BD's compressive and flexural strength surpasses that of MTA. Its high biocompatibility and excellent bioactivity enhance its appeal further as a dental material [ 9 - 10 ].

In addition to a proper pulpotomy procedure, the seletion of an appropriate restoration is a key factor in the long-term success of the treatment [ 11 ]. These teeth have been shown to have lower strength compared to intact teeth, mainly due to the preparation of access cavities and the removal of marginal ridges and pulpal roofs. 

In a review of clinical studies, these teeth were typically restored using amalgam or composite resin restorations and crowns [ 6 , 11 - 12 ]. A systematic review comparing amalgam and composite restoration after pulpotomy, in terms of clinical outcomes and survival analysis, revealed no significant difference in performance, with some studies showing better results for amalgam than composite resin [ 6 ]. In the mentioned survival analysis, some cases of failure were attributed more to tooth fracture than to the failure of the pulpotomy treatment itself. The incidence of tooth fracture in relation to the final restoration showed an advantage for crown restoration, followed by amalgam; the poorest outcomes were reported with composite resin restoration [ 6 ]. In Amend *et al*. study [ 13 ], a higher late failure rate for amalgam restoration than composite resin restoration in pulpotomized teeth was reported. A systematic review on clinical effectiveness of restorative materials after pulpotomy of primary teeth concluded that amalgam had the highest failure rate followed by compomer, open sandwich technique with resin-modified glass-ionomer (RMGI) plus composite resin and composite resin [ 13 ]. In a recent clinical study, stainless steel (ss) crown revealed a higher one-year survival rate in pulpotomized primary molar compared to bulk-fill glass-ionomer [ 14 ].

However, adhesive composite restorations are capable of bridging between the buccal and lingual cusps of weakened teeth, thereby improving their fracture resistance (FR). This intracoronal strengthening is particularly notable when the loss of tooth structure is less than 2/3 of the intercuspal distance [ 2 , 11 ]. 

Considering the uncertain results associated with vital pulp treatment in specific patients, selecting a direct and economical restoration using amalgam or composite resin, which offers sufficient strength to endure masticatory forces, seems to be a more pragmatic option than crown restorations [ 6 ]. 

On the other hand, polymerization shrinkage stresses remain the most significant problem associated with composite resins. This issue can lead to problems in marginal adaptation and subsequently microleakage, as well as recurrent caries, especially at the cervical margin of proximal boxes [ 15 - 16 ]. To mitigate the side effects of polymerization shrinkage, various techniques have been proposed. One such approach involves the use of an intermediary layer like resin-modified glass ionomer (RMGI), which is effective in reducing microleakage due to its chemical adhesion to enamel and dentin, leading to a better seal [ 17 - 19 ].

Another method for restoring teeth with adhesive material is the application of low-shrinkage composite resin with a 4mm thickness depth of cure, known as bulk-fill composites [ 15 ]. This technique offers the benefits of reduced treatment time and a lower risk of air entrapment and moisture contamination [ 20 ]. Conventional composite should be inserted incrementally to reduce shrinkage stress and provide sufficient curing of each layer [ 21 ].

Given the advantages and disadvantages of amalgam and two types of composite restorative techniques including conventional and bulk fill resin composites, clinicians face a challenge in selecting a post-pulpotomy restorative treatment that ensures sufficient fracture resistance for the restored teeth. The comparison between BD and MTA in association with the three restorative techniques (amalgam and two types of composite resin) has not been investigated in the past studies. Consequently, the purpose of this study was to evaluate the impact of different restorative techniques on the fracture behavior of premolars following MTA/BD-pulpotomy. The null hypothesis of this study was that there would be no difference in the fracture resistance/mode of MTA and BD-pulpotomized premolars restored with various materials. 

## Materials and Method

The sample size was calculated based on a previous study [ 22 ], which specified that the sample size for each group should be at least ten, with a desired statistical power of 0.80, a significance level of 0.05, and an effect size of 0.723. According to the mentioned study, each group included 10 samples. A total of ninety single-rooted, intact human maxillary premolar teeth of similar dimensions (mesiodistal dimension: 7.4±0.5; buccolingual dimension: 9.3±0.5) were included, extracted for orthodontic reasons. Selected teeth were cleaned and examined under a light microscope (Micron DPTIK, Micron Instrument Industries, India) at 20× magnification for any existing crack or fracture [ 22 ]. The specimens were stored in 0.5% chloramine solution for 24 hours and in distilled water next.

In the next step, they were embedded in a cylinder of self-curing acrylic resin up to 1mm apical to the cementoenamel junction (CEJ) with the long axis of the teeth perpendicular to the base of the block. The teeth were randomly divided into 9 groups (n= 10).

In group 1 (intact/control), teeth were kept intact and served as control. For the remaining eight groups, class II mesio-occluso-distal (MOD) cavities were prepared with the gingival cavosurface margin located 1 mm above the CEJ. The buccolingual width of each cavity was measured by using a digital caliper (Mitutoyo, Corp, Kawasaki, Japan). The facial and lingual walls of the occlusal segment were prepared parallel to each other. Endodontic access cavities were prepared with a high-speed bur under constant water cooling, and a complete coronal pulpotomy procedure was conducted.

**Figure 1:**
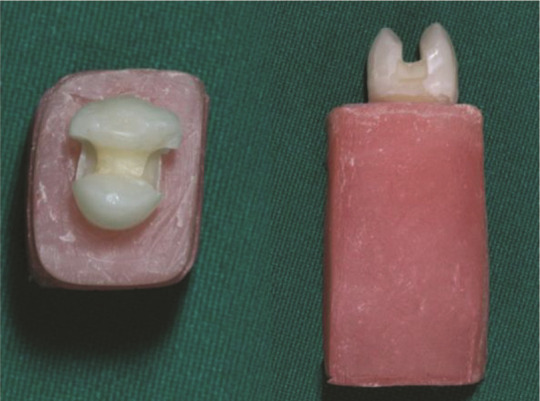
Biodentine (BD)-Pulpotomized sample

The samples were divided into two subgroups (4, 6, and 8) and (5, 7, and 9) according to the type of the pulpotomy material used, namely MTA and BD, respectively. Gray ProRoot MTA (Dentsply Tulsa Dental Specialties, Tulsa, OK, USA) and BD (Septodont, Saint Maur des Faussés, France) were prepared per the manufacturer's instructions. A 3-mm thick layer of MTA or BD was placed over the amputated dentinal walls over canal orifices in the pulp chamber using an MTA carrier (Dovgan MTA Carrier 0.9 mm bendable, Quality Aspirators, TX, USA), as demonstrated in Figure 1, and covered with a wet cotton pellet. Then the cavity was restored with a temporary restoration (Cavit, 3M ESPE, Seefeld, Germany).Following MTA insertion, the remaining cavity depth was approximately 5.5 mm. 

After one week, the cotton pellets were removed, and the restorative procedures were continued as follows. For groups 2 (MTA/unrestored) and 3 (BD/ unrestored), the samples were not restored after MOD cavity preparation and MTA or BD placement. For groups 4 (MTA+GI/Am) and 5 (BD+GI/ Am), a 2 mm-layer of conventional glass ionomer (GI) (Fuji II, GC, Japan) was applied on set MTA/ BD, and upon setting, the cavity was filled with high copper amalgam in 3.5mm thickness (ANA 2000, Nordiskd, Dental AB, Angelholm, Sweden). For groups 6 (MTA+RMGI/ conventional com) and 7 (BD+ RMGI/ conventional com), a 2mm thick layer of RMGI (Fuji II LC, GC, Japan) was applied on set MTA/BD.

The cavity surfaces were etched with 37% phosphoric acid (Denfil Etchant, Vericom Co, Georggi, Korea) for 15 seconds, rinsed for 20 seconds, and gently air-dried. Next, two consecutive coats of bonding agent (Adper Single Bond, 3M, USA) were applied and gently air-dried for 2 to 5 seconds and light-cured with a halogen light-curing device (Elipar, Paradigm, 3M, United States) at 650mW/cm2 light intensity for 20 seconds.

The conventional composite resin (Z250, 3M, USA) was inserted in two layers with 1.75 mm thickness, and each layer was cured for 20 seconds.

**Figure 2:**
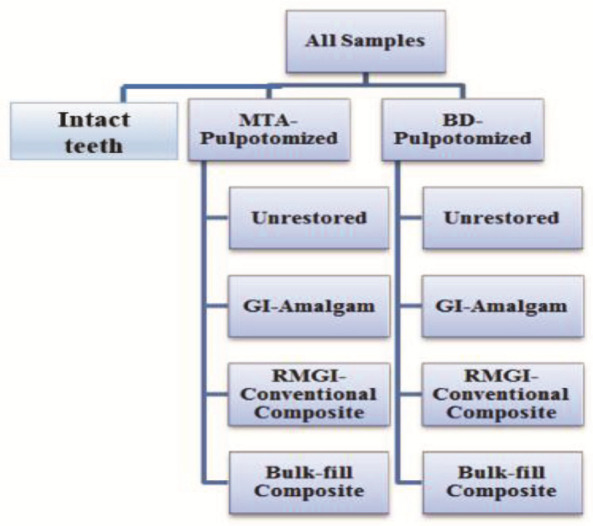
mineral trioxide aggregate (MTA); Biodentine (BD); glass ionomer (GI), resin-modified glass ionomer (RMGI)

For groups 8 (MTA+bulk-fill com) and 9 (BD+ bulk-fill com), following the adhesive procedures outlined previously, a 2-mm layer of bulk-fill flowable composite (X-tra base, Voco, Germany) was applied. This layer was then light-cured for 20 seconds, after which it was covered with a layer of bulk-fill high-viscosity composite resin (X-tra fill, Voco, Germany) with 3.5mm thickness, which was also light-cured for 20 seconds. The study groups are shown in Figure 2. 

The materials used in this study are shown in Table 1. 

**Table 1:** Materials and their composition

Materials	Manufacturer	Country	Composition
ProRoot MTA	Dentsply Tulsa Dental Specialties	USA	Powder: tricalcium silicate (CaO)_3_ · SiO_2_, dicalcium silicate (CaO)_2_ · SiO_2_, tricalcium aluminate (CaO)_3_ · Al_2_O_3_, bismuth oxide Bi_2_O_3_, gypsum CaSO_4_ · 2 H_2_O
			Liquid: distilled water H_2_O
Biodentine	Septodont	France	Powder: tricalcium silicate Ca_3_SiO_5_ (>70%), dicalcium silicate Ca_2_SiO_4_ (<15%), zirconium oxide ZrO_2_ (5%), calcium carbonate CaCO_3_ (>10%), iron oxides (<1%)
			Liquid: water H_2_O, calcium chloride CaCl_2_ (>15%), hydrosoluble polymer (polycarboxylate)
Adper Single Bond	3M ESPE	USA	Bis-GMA; HEMA;Dimethacrylate; Polyalkenoic acid copolymer; initiators; water; and ethanol
Conventional GI, Fuji II	GC Corporation	Japan	Powder: fluoroaluminosilicate glass
			Liquid: polyacrylic acid, itaconic acid, tartaric acid, maleic acid, water
RMGI, Fuji II LC	GC Corporation	Japan	Powder: fluoroaluminosilicate glass
			Liquid: polyacrylic acid, 2-hydroxyl ethyl methacrylate, urethane dimethacrylate, camphorquinone, distilled water
Amalgam, ANA2000	Nordiska Dental	Sweden	
Conventional Composite resin, Filtek Z250	3M ESPE	USA	Matrix: Bis-GMA, Bis-EMA, UDMA, CQ, Zr/Si fillers
Bulk-fill composite resin, X-tra base	Voco	Germany	Bis-EMA 10-25%, aliphatic dimethacrylate 10%25%

**Figure 3:**
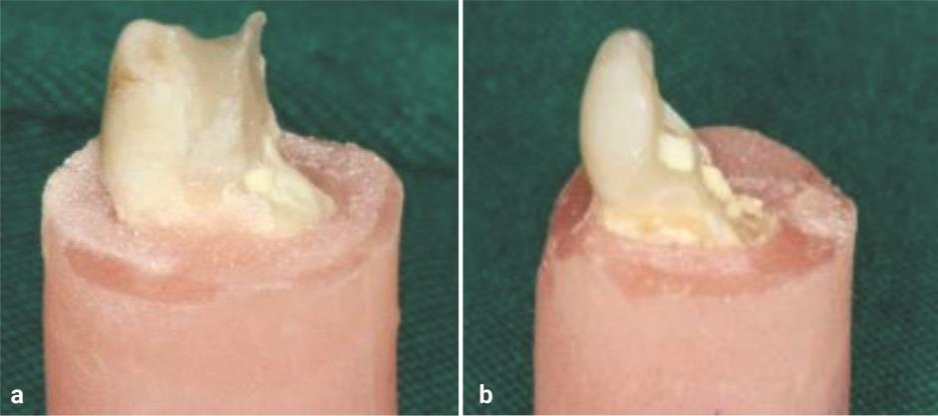
The samples of restorable/ non-restorable sample fractures, **a:** Restorable fracture, **b:** Non-restorable fracture

The teeth were stored in distilled water for 24 hours at 37ºC. In the next step, using a universal testing machine (Zwick Roell, Ulm, Germany), a compressive force was exerted by a stainless steel ball at a cross-head speed of 1 mm/min parallel to the longitudinal axis of the tooth, so that the ball was in contact with buccal and lingual cusps until fracture occurred. The force was calculated in Newton (N) at the moment of fracture as fracture resistance. The fractured specimens were evaluated to determine the fracture mode. The fracture modes were classified as mode 1; restorable, in which the end of the fracture line was at or above the CEJ, mode 2; non-restorable, in which the fracture line was more than 1 mm below the CEJ (Figure 3). 

The data were analyzed using one-way ANOVA followed by the post-hoc Tukey test, using SPSS (SPSS V21 INC, Chicago, IL). The significant difference was set to 0.05. 

## Results

Table 2 presents mean FR values with standard deviations for all study groups in Newton (N), in addition to fracture mode. When comparing the two MTA/ and BD/ unrestored groups, although the latter (group 3) revealed a higher value of resistance, this difference was not significant (*p*> 0.05). The highest FR belonged to the control (intact) group, which was statistically significant compared to all others (*p*< .05). Group 2 (MTA/ unrestored), with the lowest FR, had significantly lower FR compared to all composite resin restored groups (6-9) (*p*< .05). However, the difference of group 2 with two amalgam restored groups (4 and 5) was not significant (*p*> .05). All restoration groups (4-9) showed comparable FR than that of BD/unrestored group (group 3). 

**Table 2:** Mean±standard deviation of fracture resistance and distribution of fracture modes in various groups, N (n=10). Means followed by different superscript letters are significantly different

Group Abbreviation	Mean	Std. Deviation	Fracture Mode Restorable/ Nonrestorable
1) Intact/Control	939.44 ^a^	114.030	9/1
2) MTA/Unrestored	273.60 ^b^	45.15	3/7
3) BD/Unrestored	391.40 ^bc^	39.424	4/6
4) MTA+GI/Am	382.40 ^bc^	54.089	4/6
5) BD+GI/Am	389.50 ^bc^	71.231	5/5
6)MTA+RMGI/conventional composite	431.40 ^c^	45.926	6/4
7)BD+RMGI/conventional composite	464.30 ^c^	167.055	7/3
8)MTA+ bulk-fill composite	444.60 ^c^	80.660	7/3
9)BD+ bulk-fill composite	492.90 ^c^	62.052	9/1

Mineral trioxide aggregate (MTA); Biodentine (BD); glass ionomer (GI); amalgam (Am); resin-modified glass ionomer (RMGI)

^a,b,c^: Means followed by different superscript letters are significantly different

Most of the samples showed restorable fracture (mode1). There was no significant difference between fracture mode rates of the restored groups (*p*> 0.05). The only significant difference was related to the groups 1 (intact) and 9 (BD/ bulk-fill) with group 2 (MTA/ unrestored) (*p*= 0.02). The samples of restorable and non-restorable fractures were shown in Figure 3. 

## Discussion

According to the findings of the present study, composite resin restorations exhibited higher values of FR compared to amalgam with a GI liner in MTA- or BD- pulpotomized premolars. However, this difference was not statically significant. Therefore, the null hypothesis tested was accepted. Furthermore, in MTA-pulpotomized teeth, the FR of GI/Am restorations were similar to that of unrestored teeth.

Several studies have demonstrated that teeth restored with amalgam tend to have lower FR compared to those restored with composite resin [ 16 , 23 - 24 ]. This may be attributed to the lack of adhesive bond to cavity walls and the reinforcing effect of amalgam [ 16 , 21 ]. Some authors have shown that restoring endodontically-treated teeth with direct composite resin results in higher FR values compared to amalgam restorations [ 25 - 26 ]. A recent study, however, which contrasts with the present study, concluded that RMGI or an adhesive base combined with composite resin resulted in higher FR than amalgam restorations in MTA-pulpotomized premolars [ 23 ]. In another FR study, no differences between amalgam, conventional composite resin and bulk-fill giomer were found in MTA or CEM cement pulpotomized premolars [ 27 ].

In our study, no significant difference in FR was observed between bulk-fill and conventional composite resin restorations. This finding was in agreement with result of the latter study as mentioned above [ 27 ]. However, Ghajari *et al*. [ 28 ] assessed FR of pulpotomized primary molar that restored with bulk-fill and conventional composite resin with incremental application technique. They reported the higher FR value for bulk-fill composite resin. Fracture resistance of endodontically-treated teeth with conventional and bulk-fill flowable composite resins has been evaluated in several studies [ 17 , 29 - 30 ]. While some studies reported less fracture in cases restored with conventional composite resin others found higher FR values with flowable bulk-fill composites [ 31 - 32 ]. However, these studies did not specifically focus on pulpotomized teeth. 

Rossato *et al*. [ 33 ] found that bulk-fill composite resin used in restoring posterior teeth with extensive MOD cavities demonstrated better FR performance. Other authors have shown that teeth restored with bulk-fill composite resin exhibited almost the same FR values as those restored with conventional composites, but with better restorability after fracture [ 33 - 34 ]. These findings are consistent with the results of our study regarding FR; however, no difference in the type of fracture mode was observed between the two types of composite resin used. These different reported results may be attributed to the type of tooth, type of adhesive system and base materials, and remaining tooth structure after cavity preparation.

Bulk-fill resin composites can be classified into two groups. One group is designed for direct exposure to the oral environment, typically with high viscosity, and the other group is intended for use as a base or liner, usually with low viscosity or flowable properties, and requiring a capping layer of conventional composite resin. Rizzant *et al*. [ 35 ] reported that all tested bulk-fill composite resins, including X-tra fil, showed lower volumetric shrinkage compared to conventional composites. The X-tra fil resin, with increased filler size and high filler content (86 wt%), demonstrated reduced light scattering and shrinkage. In another study, analysis revealed that X-tra base bulk-fill composite resin exhibited the lowest polymerization shrinkage among the alternatives [ 36 ].

Furthermore, other mechanisms may contribute to decreasing the contraction tensions of bulk-fill composite resins, such as the introduction of new monomers and rheological modulators like urethane dimethacrylate, reducing polymerization stresses. The differences in polymerization contraction gaps among various composite resins are related to their significantly different filler volumes, as observed in the X-tra base resin, which shows smaller gaps and 75% filler particles per volume, a notably higher value than other bulk-fill composites [ 37 - 38 ]. 

Another notable finding of the current study was that although composite resin, particularly the bulk-fill type, displayed a higher value of FR than the unrestored group in BD-pulpotomized premolars, this difference was not significant. FR of both amalgam and composite resin restored teeth did not significantly differ from BD/ unrestored teeth. However, a similar observation was not detected in the MTA groups. This finding revealed the important role of BD as an adequate pulpotomy material. In the studies conducted by Subash D *et al*. [ 39 ] and Kumbaiah *et al*. [ 37 ], the effect of BD on the FR of endodontically treated teeth was evaluated. The former used BD as a core material in central incisors, and the latter used BD as a base material in endodontically treated premolars. These studies demonstrated a higher number of restorable fractures with BD. Although the positive correlation between FR and bond strength is not obvious, it is generally accepted that the successful bond of dental materials to root dentin increases its reinforcing effect. Subash D *et al*. [ 39 ] reported that BD releases higher amounts of calcium compared to conventional MTA, and revealed a higher depth of incorporation into dentin. These advantages may result in a stronger formation of mineral infiltration layer and tag-like structures at the dentin interface [ 40 ]. This superior interaction could contribute to the increased bonding ability of BD [ 41 ] and subsequently increase the reinforcing effect of BD [ 42 ]. However, in the current study no significant difference between FR and fracture mode of MTA and BD restored groups was obtained. Contrary to our findings, a recent study showed that the FR of pulpotomized primary molars restored with composite was higher when using BD compared to MTA [ 43 ]. 

Furthermore, compared to MTA, BD showed enhanced physical, mechanical, and handling properties, and similar (or even better) biological properties, making it a recommended restorative material for replacing dentin. BD has a modulus of elasticity similar to that of dentin, leading to stress distribution in endodontically treated roots, and reducing the risk of vertical fracture [ 44 ]. 

The load of mastication on maxillary premolars is 179 to 400 N [ 45 ]. In the present study, teeth restored with either composite resin after pulpotomy showed FR of more than 400 N, with the highest value for BD-pulpotomized and bulk-fill composite restored teeth, which makes the composite restorations the restoration of choice after MTA- or BD- pulpotomy.

In the current study, restorable fracture was the main fracture in the composite restorations. The restorable fracture could be as important as a high FR value in clinical practice because, in the case of fracturing of the restored teeth, the practitioner can restore the teeth. If this occurred after the success of the final prognosis of pulpotomy treatment, indirect cusp capping or crown restorations could be recommended.

The current study was conducted in extra-oral condit ions, without simulating supporting tissue. The compressive loading test is commonly used to evaluate the reinforcing ability of restorative materials. Moreover, although the axial loading could simulate physiologic function to some degree, this test with single load to fracture provides limited information about the stress distribution in tooth structure during load application. Further studies should be conducted to consider the effect of thermo-mechanical cycling and aging on the fracture strength of the restored teeth. Three-dimensional finite element stress analysis may provide more comprehensive insights into this issue. While evaluating the stresses generated within the tooth structure, the combination of destructive mechanical tests with nondestructive analysis such as finite element simulation may be more valuable. 

## Conclusion

According to the finding of the present study, FR of all the restoration groups was lower than intact teeth group. Conventional and bulk-fill composite resin restorations can increase the fracture resistance of premolars after MTA-pulpotomy. This increase was not significant in BD-pulpotomized premolars. There was no significant difference between restoration groups. The bulk-fill composites have beneficial properties, such as time-saving application, good adaptation with the tooth surface in case of flowable bulk-fill composite and suitable aesthetics. Therefore, they can be recommended for use in premolars after MTA- or BD-pulpotomy treatment.
